# Effects of chlorine dioxide on the germination, oxidative metabolism and growth of barley seedlings (*Hordeum vulgare* L.)

**DOI:** 10.1038/s41598-019-42295-7

**Published:** 2019-04-08

**Authors:** Ruiming Wang, Bingcui Chen, Tengfei Wang, Piwu Li, Feng Ding

**Affiliations:** Institutional affiliations: State Key Laboratory of Biobased Material and Green Papermaking (LBMP), Shandong Provincial Key Laboratory of Microbial Engineering, College of Food Science and Engineering, Qilu University of Technology (Shandong Academy of Sciences), Jinan, 250353 P.R. China

## Abstract

The effects of chlorine dioxide, ClO_2_, on the germination, oxidative metabolism and growth of barley seedlings were investigated. Barley seeds were separately treated with 0, 500, 1000 and 2000 mg.L^−1^ ClO_2_ solutions. Differences in the percentage of seed germination were observed in treatments with 1000 and 2000 mg.L^−1^ ClO_2_ solutions only. However, 1000 and 2000 mg.L^−1^ ClO_2_ significantly decreased the germination percentage. No significant difference in the MDA content, electrolyte leakage and amount of chlorophyll was observed in seedlings germinated from seeds treated with 0, 500 and 1000 mg.L^−1^ of ClO_2_. Similarly, POD and CAT activities showed no significant differences in seedlings germinated from seeds treated with 0 and 500 mg.L^−1^ while with 1000 mg.L^−1^ ClO_2_ there was an increase of these activities. Although there was no significant difference in the above ground part fresh weight between barley seedlings in which seeds were treated with distilled water and ClO_2_, the fresh weight of barley roots in which seeds were treated with ClO_2_ was significantly higher than that of control. The total length of barley roots and the number of roots were also increased. The lignin content of barley roots was markly reduced. Staining with Evans blue indicated that barley roots were not obviously damaged. Furtherly, the stimulation of the cell membrane H^+^-ATPase activity and root activity were observed to be induced by ClO_2_.

## Introduction

Different microorganisms can contaminate barley from field through storage^1^. The malting and brewing industries are reluctant to accept mycotoxin-contaminated grain because of concerns over public safety, public perception, and product quality^2–5^. Studies have revealed that the growth of Fusarium during the malting process can result in mycotoxin production and can affect the germinative capacity and malting characteristics of the barley^1,6^. Postharvest controls have focused on chemical, physical, and biological approaches with limited success^3,5,6^. Two chemical agents of interest for reducing microorganisms in malting barley include ozone and hydrogen peroxide. These chemicals are particularly interesting to maltsters because they would not leave chemical residues in finished malt^7^. Ozonation could inactivate fungi, with mycelia being more susceptible than spores, while maintaining germination if the dosage was not too high in barley at these moisture contents^8^.

Chlorine dioxide (ClO_2_) has long been known to have fungicidal, bactericidal and viricidal properties, which inactivate a wide range of microorganisms effectively, as shown by numerous studies^9^. For this reason, ClO_2_ has widely been applied in many fields such as quarantine procedures, medical, agricultural, and industrial sterilization measures, food preservation, etc. Its usage in sanitizing procedures of fruit and vegetables is recommended by the WHO, World Health Organization, and therefore legally permitted in several countries, e.g. China, USA (Ministry of Health of the People’s Republic of China 2008; USFDA 2010). Resistance to ClO_2_ has generally increased in different groups of microorganisms, e.g. Gram-negative and Gram-positive bacteria, yeasts, molds^10^. 120 min application of 8.0 mg.L^−1^ ClO_2_ was shown to be effective in reducing yeast and mold populations in blueberries, raspberries and strawberries^9^.

However, the inconsistent effects of chlorine dioxide on the germination and growth of barley seedlings has seemed to restrict its application in the food and malting industries. The main goal of this study was to evaluate ClO_2_ effects of on the germination, oxidative metabolism and growth of barley seedlings (*Hordeum vulgare* L.).

## Materials and Methods

### Treatment with chlorine dioxide

Seeds from Australian barley (*Hordeum vulgare* L. cv. gairdner) were sterilized with 0.1% HgCl_2_ for 10 min and subsequently rinsed 3 times with distilled water. They were subsequently immersed in 0, 500, 1000 and 2000 mg.L^−1^ ClO_2_ solutions for 30 min at 25 °C in the dark. Then they were washed in flowing distilled water for one minute. Afterwards, seeds were left to germinate in cell culture dishes in a sprouting machine (DYJ-S6365, Bear) at 25 °C constant temperature. During that time, they were sprayed once every hour for one minute with distilled water that was renewed every day. At this stage, the numbers of seeds germinated was recorded daily and the percentage of germination was calculated after seeds were germinated for seven days. From each treatment, a sample of seedlings was taken to determine POD and CAT activities, leaf chlorophyll content, MDA concentration, permeability of the cell membrane, and death of root cells, root system architecture, root activity, and lignin content in roots.

### Membrane permeability and MDA analysis

Permeability of the cell membrane was determined by electrolyte leakage^11^. Conductivity was measured with a conductivity meter (Model SG3-ELK, METTLER).

MDA content was analyzed following Wang *et al*. (2016) using the following formula^12^:

$$MDA\,(mmol.k{g}^{-1})=\frac{[6.45\times (O{D}_{532}-O{D}_{600})-0.56\times O{D}_{450}]\times {V}_{t}\times {V}_{r}}{{V}_{s}\times m}$$

in which m, V_r_, V_s_, and V_t_ stand for: sample mass, total volume of the mixture in where the reaction occurred, extract volume within the mixture volume, and entire extract volume, respectively.

### Leaf chlorophyll content

The concentration of photosynthetic pigments following Xiao and Wang^13^ were determined according to:

$${C}_{t}\,(mg.{l}^{-1})={C}_{a}+{C}_{b}=18.08\times {A}_{649}+6.63\times {A}_{665}$$

where C_a_, C_b_, and C_t_ represent the concentrations of: chlorophyll a and b; and total chlorophyll, respectively. Leaf chlorophyll content was determined according to:

$$C=\frac{C(Chlorophyll\,Concentration)\times V(Extraction\,Volume)\times N(Dilution\,Multiple)}{W(Fresh\,Weight)}$$

### Antioxidant enzyme activity

For determining POD and CAT activities, 0.5 g tissue samples (fresh weight) were ground in 6 mL of 0.1 M ice-cold sodium phosphate buffer (pH 7.8). Peroxidase (POD) activity was determined according to Keren-Keiserman *et al*.^14^ and catalase (CAT) activity was measured according to Ali *et al*.^15^.

### Detection of chlorine dioxide induced root cells death by Evans blue staining

Cell death induced by ClO_2_ or damaged cells, caused by losing plasma membrane integrity, was observed using Evans blue staining^16^ with slight modifications. Barley roots were washed for 5 minutes in running water gently, after which they were stained with 0.25% Evans blue during 30 minutes. Afterwards, root samples washed again in running water for 10 minutes and then photographed.

### Detection of chlorine dioxide induced proton extrusion of barley roots

The exuding activity of proteoid roots was determined according to Feng *et al*.^17^. To avoid damage, roots were pressed into agar carefully. For visualizing the rhizosphere acidification, an incubation period of 5 hours in the dark was made in a growth chamber.

### Measurement of root system architecture

Root system architecture parameters were determined using WinRhizo Ver 5.0 (Regent Instruments, Quebec, Canada).

### Root activity determination

Root activity was measured using the TTC (triphenyl tetrazolium chloride) method. 0.5 g root samples submitted to different ClO_2_ concentrations, immersed in a 10 ml beaker with 0.4% TTC and 66 mM phosphate buffer (pH = 7.0) solution, were kept at 37 °C for 2 h. 2 ml 1 M H_2_SO_4_ were added to end the reaction. Samples, carefully wiped and cut into segments, were then put into graduated test tubes with a plug. 10 ml of methanol were added to immerse the root tip segments completely. Afterwards, the test tubes were left at 30~40 °C for 4 to 6 h in an incubator until the apical section turned completely white. Using a spectrophotometer for 485 nm colorimetry, the reducing strength of TTC was determined following:

$$TTC\,Reducing\,Strength=\frac{TTC\,Reducing\,Amount\,(\mu g)}{Root\,Sample\,Weigth\,(g)\times Time(h)\,}$$

### Lignin determination

Lignin was quantified following a method of Ding^18^. The absorbance at A280 was measured and lignin was expressed as A280/g fresh weight.

## Results

### Effects of chlorine dioxide on seed germination

**Figure 1:**
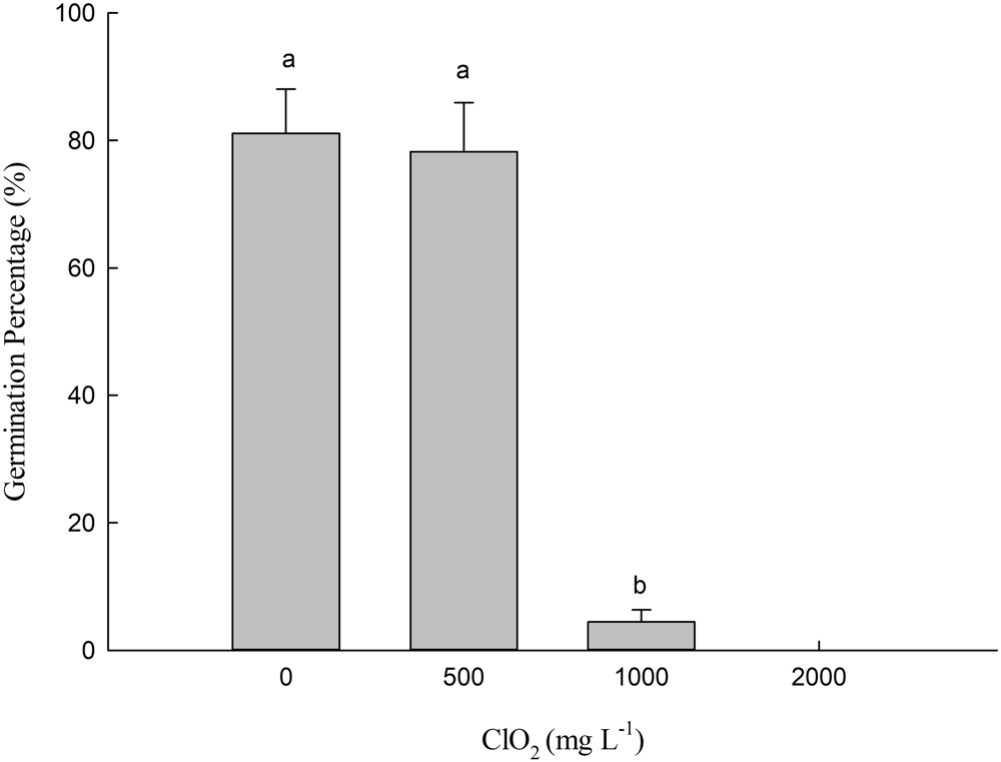
Effects of ClO_2_ on the germination percentage of barley seeds. Barley seeds were subjected to 0, 500, 1000 and 2000 mg.L^−1^ ClO_2_ solutions. Then the seeds were washed and germinated in a sprout machine for 7 days. Means with different letters are significantly different at P <0.05. Vertical bars represent standard deviations (n = 3).

The percentage and index of germination of barley seeds were significantly reduced after treatment with ClO_2_ (Fig. 1). Treated with 1000 mg.L^−1^ ClO_2_, the germination percentage decreased 94.5% compared with control (0 mg.L^−1^ ClO_2_ (CK)). However, no significant differences were observed in the percentage of seeds germinated between seeds treated with 500 mg.L^−1^ ClO_2_ and non-treated ones. At 2000 mg.L^−1^ ClO_2_, the germination percentage of barley seeds decreased to zero.

### Effects of chlorine dioxide on the fresh weight and root growth of barley seedlings

**Figure 2:**
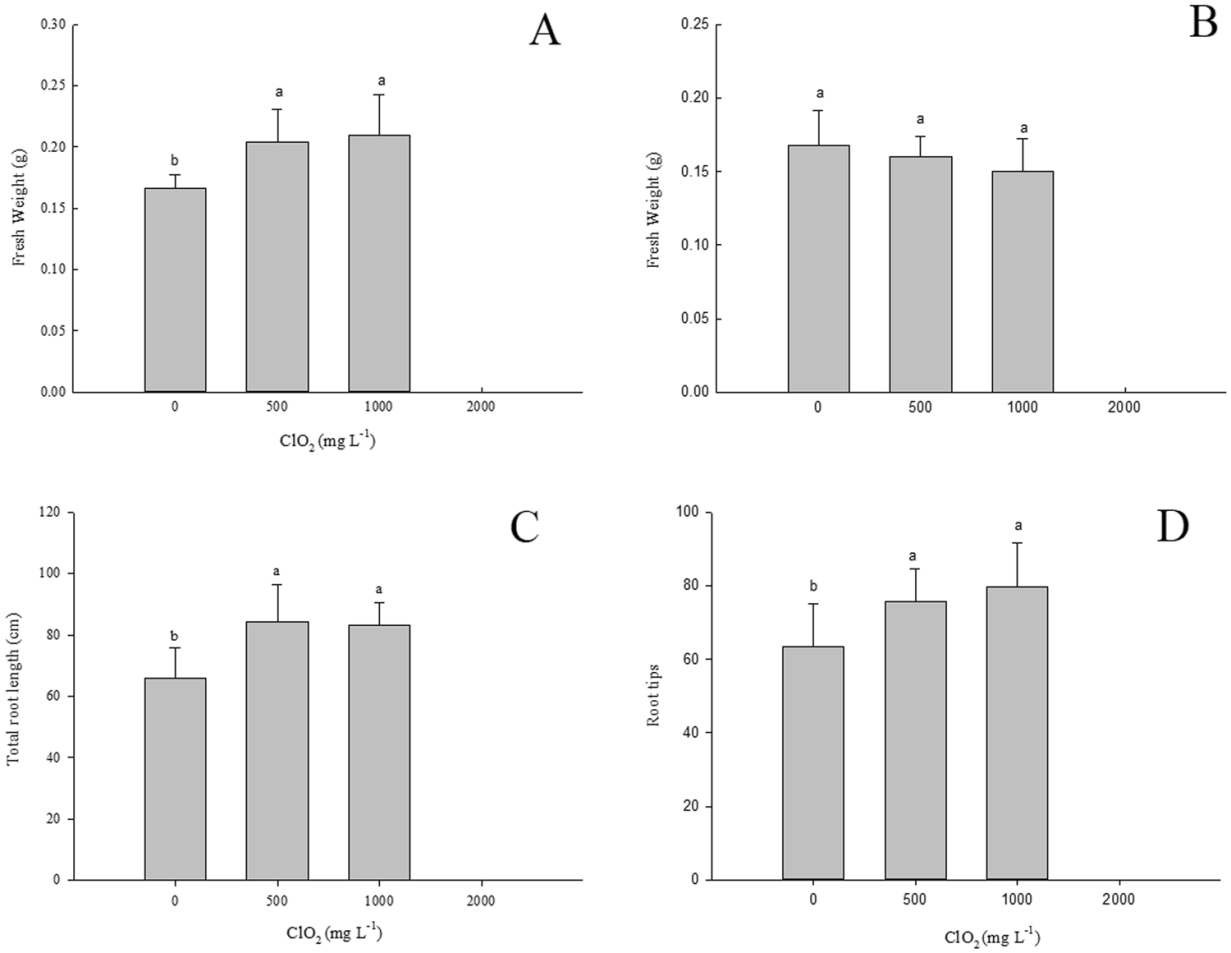
Effect of ClO_2_ on the fresh weight and root growth of barley seedlings. (**A**) Fresh weight of roots. (**B**) Fresh weight of the above ground part. (**C**) Total root length. (**D**) Number of root tips). Barley seeds were subjected to 0, 500, 1000 and 2000 mg.L^−1^ ClO_2_ solutions. Then the seeds were washed and germinated in a sprout machine for 7 days. Means and vertical bars are clarified in Fig. 1.

It was interesting to note that when seeds were treated with 500 and 1000 mg.L^−1^ ClO_2_, the fresh weight of barley roots was markedly greater than that treated with 0 mg.L^−1^ ClO_2_ (Fig. 2A). However, there was no significant difference in the fresh weight of roots between barley seedlings from seeds treated with 500 and 1000 mg.L^−1^ ClO_2_. The fresh weight of barley roots germinated from seeds treated with 500 and 1000 mg.L^−1^ ClO_2_ increased 22.9% and 26.5% respectively compared with seeds treated with 0 mg.L^−1^ ClO_2_. However, there was no significant difference in the fresh weight of above ground part among barley seedlings from seeds treated with different levels of ClO_2_ (0~1000 mg.L^−1^).

When seeds were treated with ClO_2_, the total length of barley roots was markedly greater than that of control (Fig. 2C). The total length of barley roots from seeds treated with 500 and 1000 mg.L^−1^ ClO_2_ increased 28.2% and 26.4% respectively compared with non-treated seeds. The same was also observed in the number of root tips between barley seedlings from seeds treated with ClO_2_ and control (Fig. 2D). The number of root tips from seeds treated with 500 and 1000 mg.L^−1^ ClO_2_ increased 19.5% and 25.8% respectively compared with non-treated seeds. However, there is no significant difference in the total length of barley roots and the number of root tips between barley seedlings in which seeds were treated with 500 and 1000 g.L^−1^ ClO_2_.

### Effects of chlorine dioxide on the cell membrane permeability and MDA content in barley seedlings

**Figure 3:**
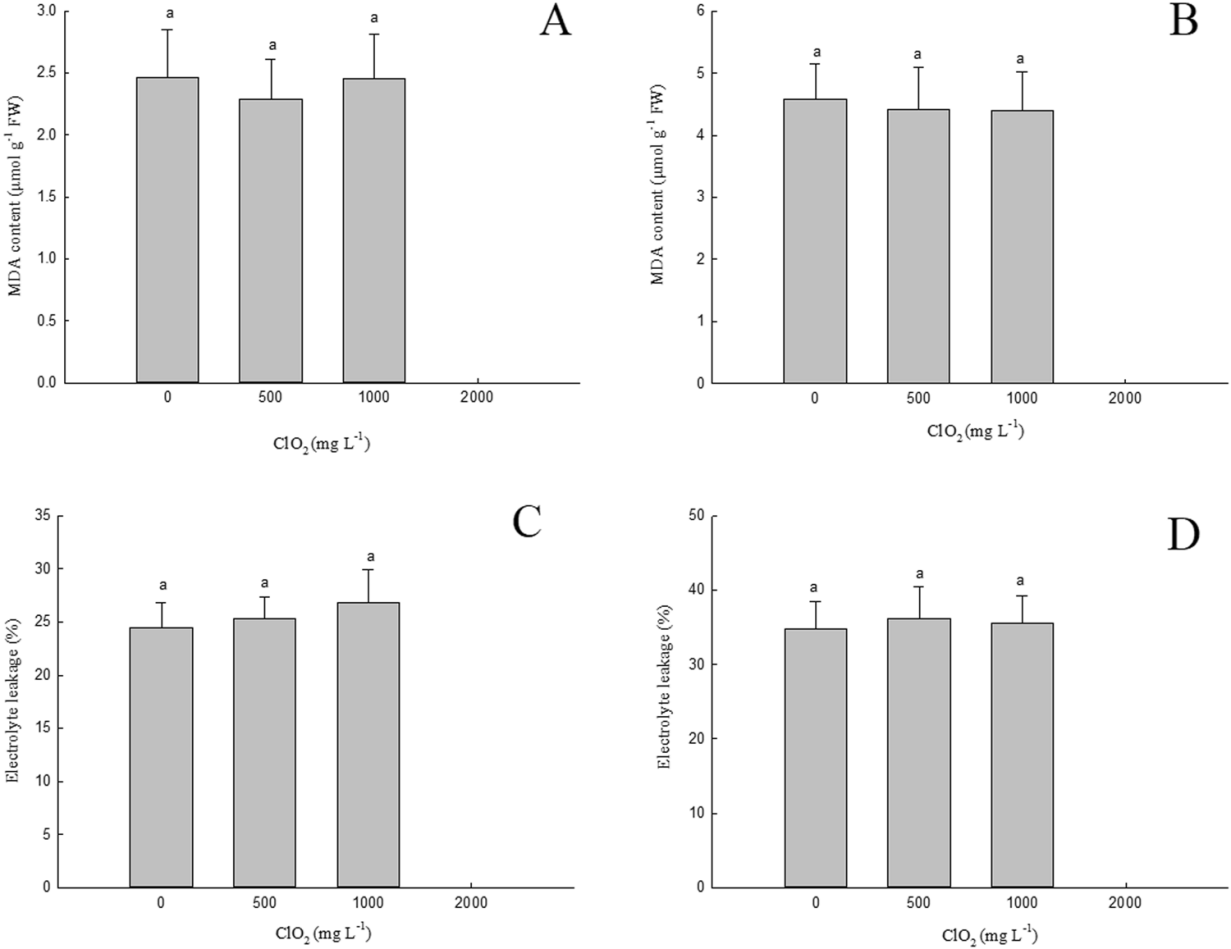
Effects of ClO_2_ on the MDA contents and electrolyte leakage of barley roots (**A**,**C**) and above ground part (**B**,**D**). Barley seeds were subjected to 0, 500, 1000 and 2000 mg.L^−1^ ClO_2_ solutions. Then the seeds were washed and germinated in a sprout machine for 7 days. Means and vertical bars are clarified in Fig. 1.

As shown in Fig. 3A,B, there were no significant differences in the concentration of MDA in barley roots and above ground part between seedlings germinated from seeds treated with 0, 500 and 1000 mg.L^−1^ ClO_2_.

In addition in Fig. 3C,D, we see that there is no significant difference in the electrolyte leakage of barley roots (C) and above ground part (D) among barley seedlings in which seeds were treated with 0, 500 and 1000 mg.L^−1^ ClO_2_.

### Effects of chlorine dioxide on the amount of chlorophyll in barley leaves

**Figure 4:**
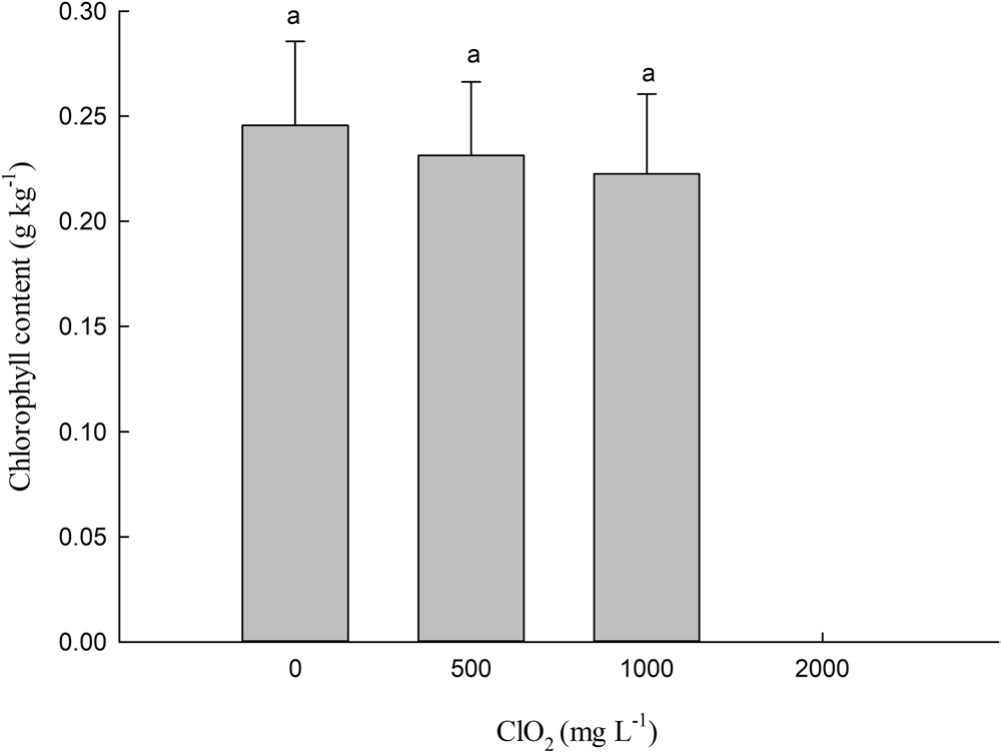
Effects of ClO_2_ on the chlorophyll content of barley leaves. Barley seeds were subjected to 0, 500, 1000 and 2000 mg.L^−1^ ClO_2_ solutions. Then the seeds were washed and germinated in a sprout machine for 7 days. Means and vertical bars are clarified in Fig. 1.

As shown in Fig. 4, no noticeable differences in the amount of chlorophyll in barley seedlings were observed in seeds treated with 0, 500 and 1000 mg.L^−1^ ClO_2_.

### Effects of chlorine dioxide on POD and CAT activities in barley seedlings

**Figure 5:**
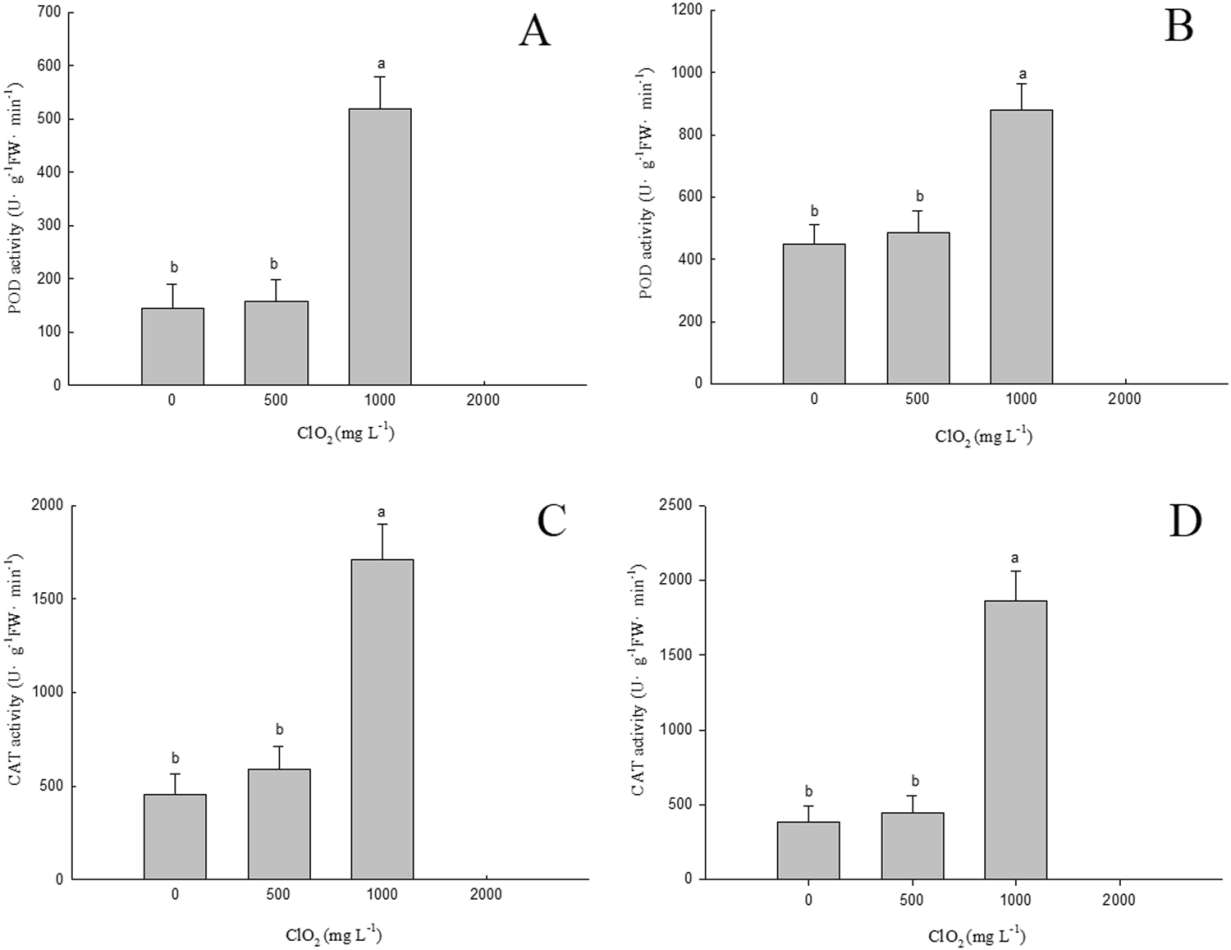
Effects of ClO_2_ on POD and CAT activities of barley roots (**A**,**C**) and above ground part (**B**,**D**). Barley seeds were subjected to 0, 500, 1000 and 2000 mg.L^−1^ ClO_2_ solutions. Then the seeds were washed and germinated in a sprout machine for 7 days. Means and vertical bars are clarified in Fig. 1.

POD and CAT activities of barley roots (A, C) and above ground part (B, D) treated with ClO_2_ were given in Fig. 5, respectively. It was shown in Fig. 5 that 1000 mg.L^−1^ ClO_2_ increased POD and CAT activities in barley roots and above ground part during seed germination. CAT activity in barley roots and above ground part germinated from seeds treated with 1000 mg.L^−1^ ClO_2_ increased 275.4% and 384.4% compared with those treated with 0 mgL^−1^ ClO_2_, respectively. POD activity in barley roots and above ground part from seeds treated with 1000 mg.L^−1^ ClO_2_ increased 260% and 96.3% compared with those non-treated (i.e. 0 mgL^−1^ ClO_2_). The data suggested that CAT and POD activities increased with high concentration of ClO_2_ treatment in barley seedlings. However, POD and CAT activities showed no significant differences in barley roots and above ground part between seeds treated with 500 mg.L^−1^ ClO_2_ and non-treated ones. It indicated that the low concentration of ClO_2_ treatment did not increase the activities of CAT and POD.

### Detection of cell death induced by chlorine dioxide in barley roots

**Figure 6:**
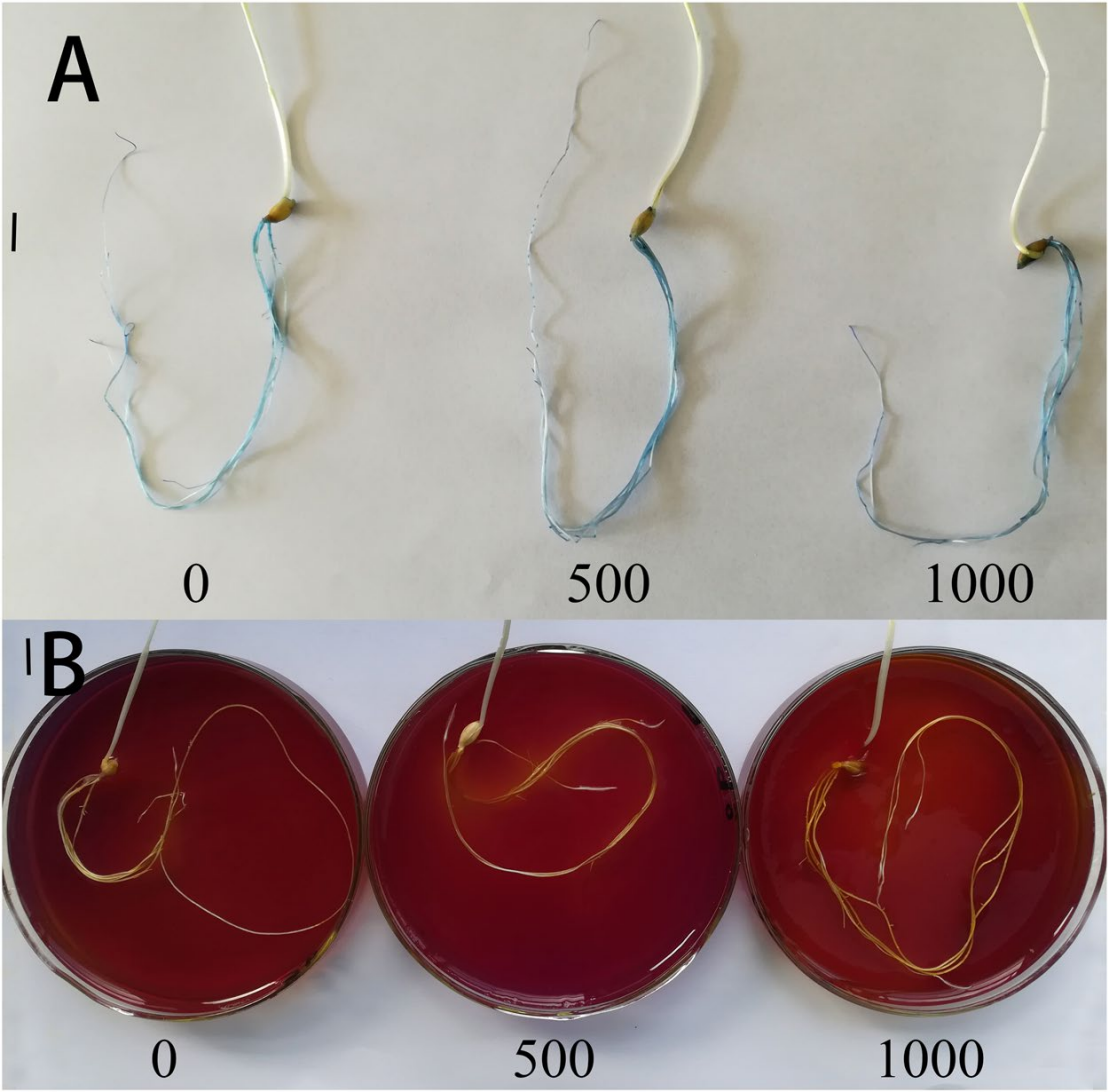
Effects of ClO_2_ on cell death (**A**) and proton extrusion (**B**) in barley roots. Barley seeds were subjected to 0, 500, 1000 and 2000 mg.L^−1^ ClO_2_ solutions. Then the seeds were washed and germinated in a sprout machine for 7 days. For (**A**), roots were stained with Evans blue for detection of ClO_2_-induced cell death; For (**B**), root samples were carefully spread on the surface of agar sheet after washing them with deionized water.

The damages caused by ClO_2_ were noticed in barley seedlings roots treated with distilled water for 7 days and stained with Evans blue. Whether seeds were soaked with ClO_2_ or not, the roots of barley seedlings were scarcely stained (Fig. 6A).

### Effects of chlorine dioxide on the cell membrane H^+^-ATPase activity in barley roots

For determining the H^+^-ATPase activity of barley roots an agar sheet with bromocresol purple, as a pH indicator, was used. Barley seedlings roots from seeds soaked with ClO_2_ showed stronger rhizosphere acidification than those soaked without ClO_2_. The roots of barley seedlings in which seeds were soaked with 1000 mg.L^−1^ ClO_2_ showed stronger rhizosphere acidification than those soaked with 0 and 500 mg.L^−1^ ClO_2_ (Fig. 6B).

### Effects of chlorine dioxide on the root activity and lignin content in barley roots

**Figure 7:**
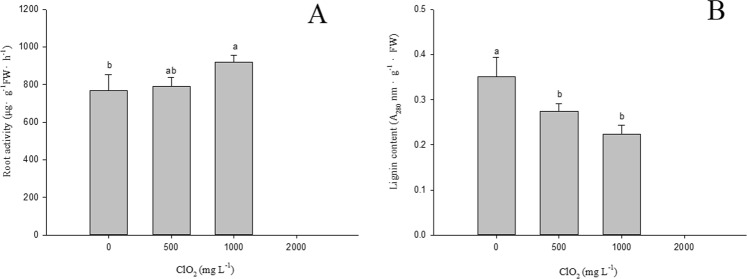
Effects of ClO_2_ on the root activity (**A**) and lignin content (**B**) in barley roots. Barley seeds were subjected to 0, 500, 1000 and 2000 mg.L^−1^ ClO_2_ solutions. Then the seeds were washed and germinated in a sprout machine for 7 days. Means and vertical bars are clarified in Fig. 1.

When seeds were treated with 1000 mg.L^−1^ ClO_2_, the root TTC reducing strength activity of barley seedlings was markedly greater than that from seeds treated with 0 mg.L^−1^ ClO_2_ (Fig. 7A). Treated with 1000 mg.L^−1^ ClO_2_, the TTC reducing strength increased 19.7% compared with 0 mg.L^−1^ ClO_2_. However, there is no significant difference in the root activity of barley seedlings between barley seedlings in which seeds were treated with 0 and 500 mg.L^−1^ ClO_2_.

The lignin content of barley roots was significantly reduced after treatment with 500 and 1000 mg.L^−1^ ClO_2_ (Fig. 7B). Treated with 500 and 1000 mg.L^−1^ ClO_2_, the lignin content of barley roots decreased 22% and 36.2% compared with 0 mg.L^−1^ ClO_2_, respectively. However, no significant differences were observed in the lignin content of barley roots between seeds treated with 500 mg.L^−1^ ClO_2_ and 1000 mg.L^−1^ ClO_2_.

## Discussion

Although there was no significant effect of 500 mg.L^−1^ ClO_2_ on the percentage of germination of barley seeds, seed germination was significantly inhibited at 1000 mg.L^−1^ ClO_2_. 1000 mg L^−1^ ClO_2_ strongly reduced germination and therefore seeds with a strong resistance could germinate. However, priming of seeds with 500 and 1000 mg.L^−1^ ClO_2_ increased the fresh weight of barley roots significantly. This may be partly caused by the increase of the total length of barley roots and the number of roots (Fig. 2C,D). ClO_2_ might deform chlorophyll in *Phaeocystis globosa* by single molecular diffusion, damage the cell membrane system by lipid peroxidation, and eventually led to the death of cells^19^. However, seed soaking with ClO_2_ did not significantly decrease leaf chlorophyll content. It indicated that there was very little ClO_2_ residue in malt.

The plasma membrane is a selective permeable boundary that involves cells and their organelles, thus controlling the exchange of substances between the interior and the surrounding environment of the cell or organelle. It transfers signals from the outside to the inside, participates in the synthesis and assembly of substances and provides physical connections for cells and substances outside^20^. It has been observed that abiotic stress can cause excess accumulation of free radicals in plant cells^21,22^. The excess free radicals thus accumulated oxidize the unsaturated fatty acids of plasma membranes^23^, leading to the peroxidation of its lipids^24,25^. Consequently, the cell membrane is damaged, its selectivity is destroyed thus increasing the leakage of electrolytes from the cytoplasm (i.e. there is an increase in membrane permeability). Therefore, MDA content and membrane permeability can be used as indicators of the cell membrane degree of injury^24,25^. CAT and POD are two major antioxidant enzymes related to the germination of plant seeds^26^. Free radicals (e.g. O^2−^, OH^−^, H_2_O_2_) induced by abiotic stress can be removed by CAT and POD effectively, thus protecting plant cells from abiotic stress damage^22^. Our experimental results show that 500 mg.L^−1^ ClO_2_ do not stimulate CAT and POD activities significantly while 1000 mg.L^−1^ ClO_2_ increases them in roots and above ground part (Fig. 5A–D) remarkably. This could be due to the residues of ClO_2_ in barley seedlings. This increase in CAT and POD activities led to an increase in the antioxidant capacity followed by a decrease of MDA concentration and permeability of the cell membrane.

Cell death induced by ClO_2_ and caused by the degradation of cell membranes was observed using Evans blue staining. Staining was not stronger in roots treated with ClO_2_ (Fig. 6A) compared with non-treated roots, thus indicating that the number of cells dead or injured did not increase during treatment with ClO_2_. These results indicate that ClO_2_ did not cause obvious damages to the cell membrane of barley roots.

The H^+^-ATPase of the cell membrane generates gradients of electric potential and pH by extruding H^+^ from the cytoplasm to the outside, thus controlling the pH of the cytoplasm and of the external immediate surrounding of plant cells directly. In order to assess if ClO_2_ could stimulate the cell membrane H^+^-ATPase activity, H^+^-release was measured in barley roots. After treatment with ClO_2_, barley roots showed stronger acidification of the rhizosphere than those treated without ClO_2_ (Fig. 6B). Root activity can reflect metabolic status of root in a certain extent and is related to the ability of plants to absorb nutrients. The root activity of barley seedlings from seeds treated with 1000 mg.L^−1^ ClO_2_ was significantly greater than that from seeds treated without ClO_2_. Cellular expansion is initiated by acidification of the external medium due to the activation of the cell membrane H^+^-ATPase^27,28^. Auxin, a hormone assumed to activate H^+^-ATPase through an unknown process, could be associated with this mechanism known as the acid growth theory. Accordingly, the acidification of the apoplast leads to a wall-loosening process^29,30^ and to the hyperpolarization of the cell membrane, which induces the uptake of K^+^ ^31^. This uptake promotes osmotic changes within cells, which allow the influx of water through the cell membrane aquaporins, thus favoring elongation of cells^32^. The lignin content of barley roots was also reduced after treatment with 1000 mg.L^−1^ ClO_2_ compared with those treated without ClO_2._ Decreased lignin content increased the cell wall plasticity and, therefore, stimulated root elongation of barley.

## Conclusions

In general, soaking seeds with moderate ClO_2_ did not inhibit the germination of barley seeds and deform chlorophyll in barley leaves. On the contrary, it could promote the growth of barley roots (the total length of barley roots and the number of roots were increased) by regulating the antioxidant enzymes, the activity of the cell membrane H^+^-ATPase, the root activity and the lignin content.
